# Supplementary material for: ARF1 compartments direct cargo flow via maturation into recycling endosomes
Source: Nat Cell Biol. 2024 Oct 4;26(11):1845–59. doi: 10.1038/s41556-024-01518-4 (PMC11567898; doi:10.1038/s41556-024-01518-4)
Supplement: Supplementary file 1 — Reporting Summary [file 41556_2024_1518_MOESM1_ESM.pdf]

nature portfolio

Corresponding author(s): Francesca Bottanelli

Last updated by author(s): Aug 20, 2024

Reporting Summary

Nature Portfolio wishes to improve the reproducibility of the work that we publish. This form provides structure for consistency and transparency in reporting. For further information on Nature Portfolio policies, see our Editorial Policies and the Editorial Policy Checklist.

Statistics

For all statistical analyses, confirm that the following items are present in the figure legend, table legend, main text, or Methods section.

n/a	Confirmed	
☐	☒	The exact sample size (*n*) for each experimental group/condition, given as a discrete number and unit of measurement
☐	☒	A statement on whether measurements were taken from distinct samples or whether the same sample was measured repeatedly
☐	☒	The statistical test(s) used AND whether they are one- or two-sided<br>*Only common tests should be described solely by name; describe more complex techniques in the Methods section.*
☐	☒	A description of all covariates tested
☐	☒	A description of any assumptions or corrections, such as tests of normality and adjustment for multiple comparisons
☐	☒	A full description of the statistical parameters including central tendency (e.g. means) or other basic estimates (e.g. regression coefficient) AND variation (e.g. standard deviation) or associated estimates of uncertainty (e.g. confidence intervals)
☐	☒	For null hypothesis testing, the test statistic (e.g. *F*, *t*, *r*) with confidence intervals, effect sizes, degrees of freedom and *P* value noted<br>*Give P values as exact values whenever suitable.*
☒	☐	For Bayesian analysis, information on the choice of priors and Markov chain Monte Carlo settings
☒	☐	For hierarchical and complex designs, identification of the appropriate level for tests and full reporting of outcomes
☐	☒	Estimates of effect sizes (e.g. Cohen's *d*, Pearson's *r*), indicating how they were calculated

Our web collection on statistics for biologists contains articles on many of the points above.

Software and code

Policy information about availability of computer code

Data collection	Microscopy data was collected on an Abberior STED microscope using the Imspector software from Abberior instruments (Version 16.3) or on a Nikon CSU-W1 SoRa spinning disk microscope using the NIS-Elements software (Version 4.50). Electron micrographs were taken with an Helios 5 CX Dual Beam (Thermo Fisher Scientific).
Data analysis	For analysis of microscopy data Fiji (ImageJ 2.7.0) was used. The JaCOP plugin was used. For aquisition and analysis of CLEM data the follwing softwares were used: Aquisition: MAPS 3 (Thermo Fisher), Analysis: Microscopy Imaging Browiser (MIB) Version 2.83 and Dragonfly (Comet Group) were used (Software version Dragonfly 2022.2). Statistical analysis was perfomed using Graphpad Prism 9.3.0. For image quantification Cell Profiler 4.2 was used . Cell Profiler pipelines are provided as Supplementary Table 5 and 6.

For manuscripts utilizing custom algorithms or software that are central to the research but not yet described in published literature, software must be made available to editors and reviewers. We strongly encourage code deposition in a community repository (e.g. GitHub). See the Nature Portfolio guidelines for submitting code & software for further information.

Data

Policy information about availability of data

All manuscripts must include a data availability statement. This statement should provide the following information, where applicable:

- Accession codes, unique identifiers, or web links for publicly available datasets
- A description of any restrictions on data availability
- For clinical datasets or third party data, please ensure that the statement adheres to our policy

All data presented in plots and diagrams is provided together with the manuscript as Source Data Files (Unprocessed Western Blots and Numerical Source Data). Microscopy data is available upon reasonable request. Light microscopy images shown in the manuscript are representative of at least 3 biological replicates.

Human research participants

Policy information about studies involving human research participants and Sex and Gender in Research.

Reporting on sex and gender	NA
Population characteristics	NA
Recruitment	NA
Ethics oversight	NA

Note that full information on the approval of the study protocol must also be provided in the manuscript.

Field-specific reporting

Please select the one below that is the best fit for your research. If you are not sure, read the appropriate sections before making your selection.

☒ Life sciences ☐ Behavioural & social sciences ☐ Ecological, evolutionary & environmental sciences

For a reference copy of the document with all sections, see nature.com/documents/nr-reporting-summary-flat.pdf

Life sciences study design

All studies must disclose on these points even when the disclosure is negative.

Sample size	No sample size calculations were performed and no statistical method was used to determine sample size. Most experiments were replicated (biological replicates) at least three times to ensure reproducibility apart from the Transferrin uptake assays in Figure 8C (n=2). The sample sizes were chosen based on experience made in previous publications/experiments (PMID: 37102998, PMID: 28428254). Microscopy images and Western Blots are shown as representative images.
Data exclusions	No data was excluded from this study.
Replication	Sample sizes are indicated in the figure legends. All replicates were successful and yielded similiar outcomes.
Randomization	Data was not randomized in this study. Randomization was not relevant as analysis of microscopy data was done without prior expectations.
Blinding	Data was not blinded in this study. Microscopy data with KI cell lines was analyzed without previous expectations. For quantification of imaging data such as Manders and Pearson correlation coefficient data was automatically analyzed with mageJ and Cell Profiler.

Reporting for specific materials, systems and methods

We require information from authors about some types of materials, experimental systems and methods used in many studies. Here, indicate whether each material, system or method listed is relevant to your study. If you are not sure if a list item applies to your research, read the appropriate section before selecting a response.

Materials & experimental systems

n/a	Involved in the study
☐	☒ Antibodies
☐	☒ Eukaryotic cell lines
☒	☐ Palaeontology and archaeology
☒	☐ Animals and other organisms
☒	☐ Clinical data
☒	☐ Dual use research of concern

Methods

n/a	Involved in the study
☒	☐ ChIP-seq
☒	☐ Flow cytometry
☒	☐ MRI-based neuroimaging

Antibodies

Antibodies used

CHC antibody: (Catalogue nr.: NB300-613, Supplier: Novus Biologicals , Clone: X22, Lot Number: UG285318)
β-Actin antibody: (Catalogue nr.: 8H10D10, Supplier: Cell Signaling , Lot Number: 20)
AP1M1 antibody: (Catalogue nr.: ab230273, Supplier: Abcam, Lot Number: GR3311789-9)
Halo antibody: (Catalogue nr.: G921, Supplier: Promega, Lot Number: 0000121291)
SNAP antibody: (Catalogue nr.: A00684, Supplier: Genscript)

Validation

CHC antibody was validated by western blot of human brain lysates, Immunocytochemistry/Immunofluorescence in HeLa, U251 and NCI-H460 Cells and Flow cytometry of HeLa cells by the supplier.
β-Actin antibody was validated by western blot of various cell lines (HeLa, COS, C2C12, C6, CHO), Immunocytochemistry/Immunofluorescence in NIH/3T3 cells and Flow cytometry of HeLa cells by the supplier.
AP1M1 antibody was validated by western blot in HeLa, Jurkat, Hep2G and A-375 cell lysates.
Halo and SNAP antibodies were both tested by the suppliers for western blot applications.

Eukaryotic cell lines

Policy information about cell lines and Sex and Gender in Research

Cell line source(s)

HeLa WT cells: ECACC General Cell Collection (93021013),
Jurkat cells: DSMZ (ACC 282)
HAP1 cells were described in Essletzbichler et. al (2014).Gift from Thijn Brummelkamp lab, Netherlands Cancer Institute

Authentication

HeLa and Jurkat cells were analysed and certificated by the supplier (HeLa: Sigma Aldric, Ref: Cancer Res 1952;12:264; Proc Soc Exp Biol Med 1954;87:480, DNA profile: Amelogenin: X, CSF1PO: 9,10, D5S818: 11,12, D7S820: 8,12, D13S317: 12,13.3, D16S539: 9,10, TH01: 7, TPOX: 8,12, vWA: 16,18, Jurkat cells: DMSZ, Statement from supplier: STR analysis according to the global standard ANSI/ATCC ASN-0002.1-2021 (2021) resulted in an authentic STR profile of the reference STR database)
HAP1 cells were not autenticated.

Mycoplasma contamination

Cell lines were not tested for any mycoplasma as gene editing was performed at low passage from supplier stock.

Commonly misidentified lines
(See ICLAC register)

We did not use any commonly misidentified cell lines.

Palaeontology and Archaeology

Specimen provenance

Provide provenance information for specimens and describe permits that were obtained for the work (including the name of the issuing authority, the date of issue, and any identifying information). Permits should encompass collection and, where applicable, export.

Specimen deposition

Indicate where the specimens have been deposited to permit free access by other researchers.

Dating methods

If new dates are provided, describe how they were obtained (e.g. collection, storage, sample pretreatment and measurement), where they were obtained (i.e. lab name), the calibration program and the protocol for quality assurance OR state that no new dates are provided.

☐ Tick this box to confirm that the raw and calibrated dates are available in the paper or in Supplementary Information.

Ethics oversight

Identify the organization(s) that approved or provided guidance on the study protocol, OR state that no ethical approval or guidance was required and explain why not.

Note that full information on the approval of the study protocol must also be provided in the manuscript.

Animals and other research organisms

Policy information about studies involving animals; ARRIVE guidelines recommended for reporting animal research, and Sex and Gender in Research

Laboratory animals	*For laboratory animals, report species, strain and age OR state that the study did not involve laboratory animals.*
Wild animals	*Provide details on animals observed in or captured in the field; report species and age where possible. Describe how animals were caught and transported and what happened to captive animals after the study (if killed, explain why and describe method; if released, say where and when) OR state that the study did not involve wild animals.*
Reporting on sex	*Indicate if findings apply to only one sex; describe whether sex was considered in study design, methods used for assigning sex. Provide data disaggregated for sex where this information has been collected in the source data as appropriate; provide overall numbers in this Reporting Summary. Please state if this information has not been collected. Report sex-based analyses where performed, justify reasons for lack of sex-based analysis.*
Field-collected samples	*For laboratory work with field-collected samples, describe all relevant parameters such as housing, maintenance, temperature, photoperiod and end-of-experiment protocol OR state that the study did not involve samples collected from the field.*
Ethics oversight	*Identify the organization(s) that approved or provided guidance on the study protocol, OR state that no ethical approval or guidance was required and explain why not.*

Note that full information on the approval of the study protocol must also be provided in the manuscript.

Clinical data

Policy information about clinical studies

All manuscripts should comply with the ICMJE guidelines for publication of clinical research and a completed CONSORT checklist must be included with all submissions.

Clinical trial registration	*Provide the trial registration number from ClinicalTrials.gov or an equivalent agency.*
Study protocol	*Note where the full trial protocol can be accessed OR if not available, explain why.*
Data collection	*Describe the settings and locales of data collection, noting the time periods of recruitment and data collection.*
Outcomes	*Describe how you pre-defined primary and secondary outcome measures and how you assessed these measures.*

Dual use research of concern

Policy information about dual use research of concern

Hazards

Could the accidental, deliberate or reckless misuse of agents or technologies generated in the work, or the application of information presented in the manuscript, pose a threat to:

No	Yes	
☐	☐	Public health
☐	☐	National security
☐	☐	Crops and/or livestock
☐	☐	Ecosystems
☐	☐	Any other significant area

Experiments of concern

Does the work involve any of these experiments of concern:

No	Yes	
☐	☐	Demonstrate how to render a vaccine ineffective
☐	☐	Confer resistance to therapeutically useful antibiotics or antiviral agents
☐	☐	Enhance the virulence of a pathogen or render a nonpathogen virulent
☐	☐	Increase transmissibility of a pathogen
☐	☐	Alter the host range of a pathogen
☐	☐	Enable evasion of diagnostic/detection modalities
☐	☐	Enable the weaponization of a biological agent or toxin
☐	☐	Any other potentially harmful combination of experiments and agents

ChIP-seq

Data deposition

☐ Confirm that both raw and final processed data have been deposited in a public database such as GEO.

☐ Confirm that you have deposited or provided access to graph files (e.g. BED files) for the called peaks.

Data access links<br>*May remain private before publication.*	*For "Initial submission" or "Revised version" documents, provide reviewer access links. For your "Final submission" document, provide a link to the deposited data.*
Files in database submission	*Provide a list of all files available in the database submission.*
Genome browser session<br>(e.g. UCSC)	*Provide a link to an anonymized genome browser session for "Initial submission" and "Revised version" documents only, to enable peer review. Write "no longer applicable" for "Final submission" documents.*

Methodology

Replicates	*Describe the experimental replicates, specifying number, type and replicate agreement.*
Sequencing depth	*Describe the sequencing depth for each experiment, providing the total number of reads, uniquely mapped reads, length of reads and whether they were paired- or single-end.*
Antibodies	*Describe the antibodies used for the ChIP-seq experiments; as applicable, provide supplier name, catalog number, clone name, and lot number.*
Peak calling parameters	*Specify the command line program and parameters used for read mapping and peak calling, including the ChIP, control and index files used.*
Data quality	*Describe the methods used to ensure data quality in full detail, including how many peaks are at FDR 5% and above 5-fold enrichment.*
Software	*Describe the software used to collect and analyze the ChIP-seq data. For custom code that has been deposited into a community repository, provide accession details.*

Flow Cytometry

Plots

Confirm that:

☐ The axis labels state the marker and fluorochrome used (e.g. CD4-FITC).

☐ The axis scales are clearly visible. Include numbers along axes only for bottom left plot of group (a 'group' is an analysis of identical markers).

☐ All plots are contour plots with outliers or pseudocolor plots.

☐ A numerical value for number of cells or percentage (with statistics) is provided.

Methodology

Sample preparation	*Describe the sample preparation, detailing the biological source of the cells and any tissue processing steps used.*
Instrument	*Identify the instrument used for data collection, specifying make and model number.*

Software | *Describe the software used to collect and analyze the flow cytometry data. For custom code that has been deposited into a community repository, provide accession details.*
Cell population abundance | *Describe the abundance of the relevant cell populations within post-sort fractions, providing details on the purity of the samples and how it was determined.*
Gating strategy | *Describe the gating strategy used for all relevant experiments, specifying the preliminary FSC/SSC gates of the starting cell population, indicating where boundaries between "positive" and "negative" staining cell populations are defined.*

☐ Tick this box to confirm that a figure exemplifying the gating strategy is provided in the Supplementary Information.

Magnetic resonance imaging

Experimental design

Design type | *Indicate task or resting state; event-related or block design.*
Design specifications | *Specify the number of blocks, trials or experimental units per session and/or subject, and specify the length of each trial or block (if trials are blocked) and interval between trials.*
Behavioral performance measures | *State number and/or type of variables recorded (e.g. correct button press, response time) and what statistics were used to establish that the subjects were performing the task as expected (e.g. mean, range, and/or standard deviation across subjects).*

Acquisition

Imaging type(s) | *Specify: functional, structural, diffusion, perfusion.*
Field strength | *Specify in Tesla*
Sequence & imaging parameters | *Specify the pulse sequence type (gradient echo, spin echo, etc.), imaging type (EPI, spiral, etc.), field of view, matrix size, slice thickness, orientation and TE/TR/flip angle.*
Area of acquisition | *State whether a whole brain scan was used OR define the area of acquisition, describing how the region was determined.*
Diffusion MRI ☐ Used ☐ Not used

Preprocessing

Preprocessing software | *Provide detail on software version and revision number and on specific parameters (model/functions, brain extraction, segmentation, smoothing kernel size, etc.).*
Normalization | *If data were normalized/standardized, describe the approach(es): specify linear or non-linear and define image types used for transformation OR indicate that data were not normalized and explain rationale for lack of normalization.*
Normalization template | *Describe the template used for normalization/transformation, specifying subject space or group standardized space (e.g. original Talairach, MNI305, ICBM152) OR indicate that the data were not normalized.*
Noise and artifact removal | *Describe your procedure(s) for artifact and structured noise removal, specifying motion parameters, tissue signals and physiological signals (heart rate, respiration).*
Volume censoring | *Define your software and/or method and criteria for volume censoring, and state the extent of such censoring.*

Statistical modeling & inference

Model type and settings | *Specify type (mass univariate, multivariate, RSA, predictive, etc.) and describe essential details of the model at the first and second levels (e.g. fixed, random or mixed effects; drift or auto-correlation).*
Effect(s) tested | *Define precise effect in terms of the task or stimulus conditions instead of psychological concepts and indicate whether ANOVA or factorial designs were used.*
Specify type of analysis: ☐ Whole brain ☐ ROI-based ☐ Both
Statistic type for inference (See Eklund et al. 2016) | *Specify voxel-wise or cluster-wise and report all relevant parameters for cluster-wise methods.*
Correction | *Describe the type of correction and how it is obtained for multiple comparisons (e.g. FWE, FDR, permutation or Monte Carlo).*

Models & analysis

n/a	Involved in the study
☐	☐ Functional and/or effective connectivity
☐	☐ Graph analysis
☐	☐ Multivariate modeling or predictive analysis

Functional and/or effective connectivity

Report the measures of dependence used and the model details (e.g. Pearson correlation, partial correlation, mutual information).

Graph analysis

Report the dependent variable and connectivity measure, specifying weighted graph or binarized graph, subject- or group-level, and the global and/or node summaries used (e.g. clustering coefficient, efficiency, etc.).

Multivariate modeling and predictive analysis

Specify independent variables, features extraction and dimension reduction, model, training and evaluation metrics.
