# Supplementary material for: ARF1 compartments direct cargo flow via maturation into recycling endosomes
Source: Nat Cell Biol. 2024 Oct 4;26(11):1845–59. doi: 10.1038/s41556-024-01518-4 (PMC11567898; doi:10.1038/s41556-024-01518-4)
Supplement: Supplementary file 16 — Unprocessed western blots. [file 41556_2024_1518_MOESM16_ESM.pdf]

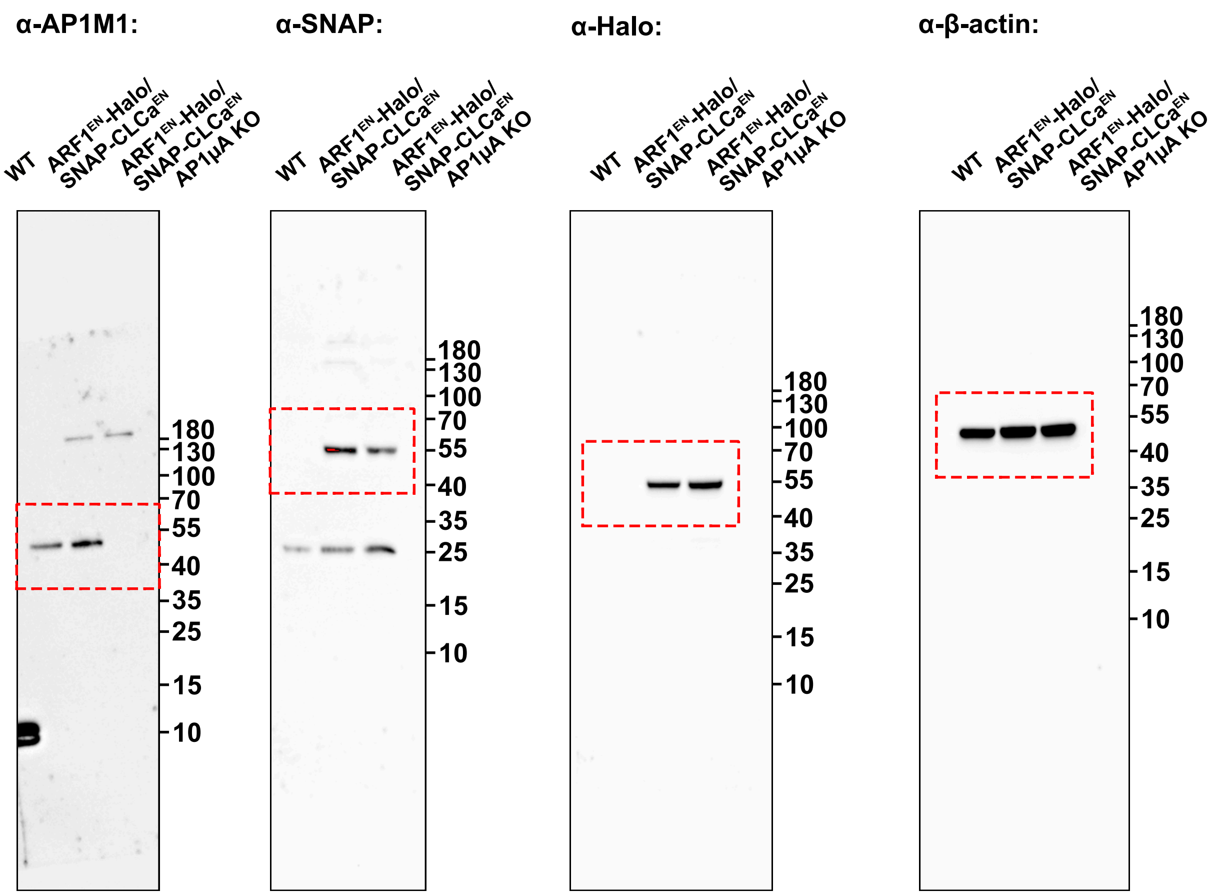
α-AP1M1:
α-SNAP:
α-Halo:
α-β-actin:
WT
ARF1EN-Halo/ SNAP-CLCaEN
ARF1EN-Halo/ SNAP-CLCaEN
AP1μA KO
180
130
100
70
55
40
35
25
15
10
